# James Baxter Bean

**Published:** 1870-10

**Authors:** 


### OBITUARY.

---

*James Baxter Bean, D.D.S,, M.D.*—The sad duty once more devolves upon us of announcing the death of an alumnus of the Baltimore College of Dental Surgery,—that of James B. Bean of this city.

By letters received at the State Department at Washington, we learn that Dr. Bean was one of the eleven unfortunate men who perished by an avalanch in attempting the ascent of Mont Blanc, in September last. He left Baltimore on a pleasure trip to Europe, in company with Dr. A. H. Balderston, his former associate in practice, about the middle of July last, and on the 13th of August, after parting with his companion at Paris, Dr. Bean went to Switzerland. A correspondent of a Boston paper writing of the disaster, thus describes the opening of some of the effects left by the unfortunate party at Chamouni previous to the fatal ascent: "Then we receive a bundle tied in a handkerchief of white linen. It is Dr. Beans. It contains his passport, made out at Washington July 12th, 1870, and forwarded to him at 58 Saratoga St., Baltimore Md. We find he is sent out by the Smithsonian Institution, and is recommended to the respectful attention and helpfulness of scientific people in Europe. There are some fine geological specimens found by him in the last few days.—There is a needle book and buttons, silk, cotton, and other endearing evidences of a woman's care. In an unsealed envelope I find, neatly sewed upon a card, a cross of white crystal. Above and below the cross are written in pencil these words: 'Top of the Bevent, (8.284 feet,) September 3, 1870. Beckie! bless your darling little heart! This shall be for you, for I thought of you as soon as I saw it."

Dr. Bean was a native of Florida, and practiced several years in the town of Micanopy, in that State, previous to entering the Baltimore College of Dental Surgery, where he graduated in 1860. After graduating, Dr. Bean practiced in Augusta and Macon, Georgia, and during the war had charge of a ward in one of the Confederate hospitals, devoted to the treatment of fractures of the jaws, holding the rank of Assistant Surgeon. A short time after the close of the war, he settled in Baltimore and succeeded in establishing a remunerative practice, in the prosecution of which he displayed untiring energy, and was highly esteemed by all who knew him.

Dr. Bean was the inventor of an Interdental Splint, composed of vulcanized rubber, which many eminent Surgeons have pronounced a valuable appliance, possessing great advantages in its complete reliability after being adjusted, being easily managed by an ordinary nurse, not easily displaced by the motions of the patient, and uniformly giving great ease and comfort.

He also invented a very complete apparatus for the manufacture and administration of Nitrous Oxide Gas, and his name became widely known in the dental profession for his valuable improvements in the manner of working Aluminum.

Dr. Bean leaves a wife and child in Jonesboro, Tenn., and for the benefit of the latter just previous to his departure from Baltimore, he effected an insurance of $10.000 on his life.

